# Supplementary material for: Tissue Sampling and Homogenization with NIRL Enables Spatially Resolved Cell Layer Specific Proteomic Analysis of the Murine Intestine
Source: Int J Mol Sci. 2022 May 30;23(11):6132. doi: 10.3390/ijms23116132 (PMC9181169; doi:10.3390/ijms23116132)
Supplement: Supplementary file 1 [file ijms-23-06132-s001.zip › Supplementary Figures.pdf]

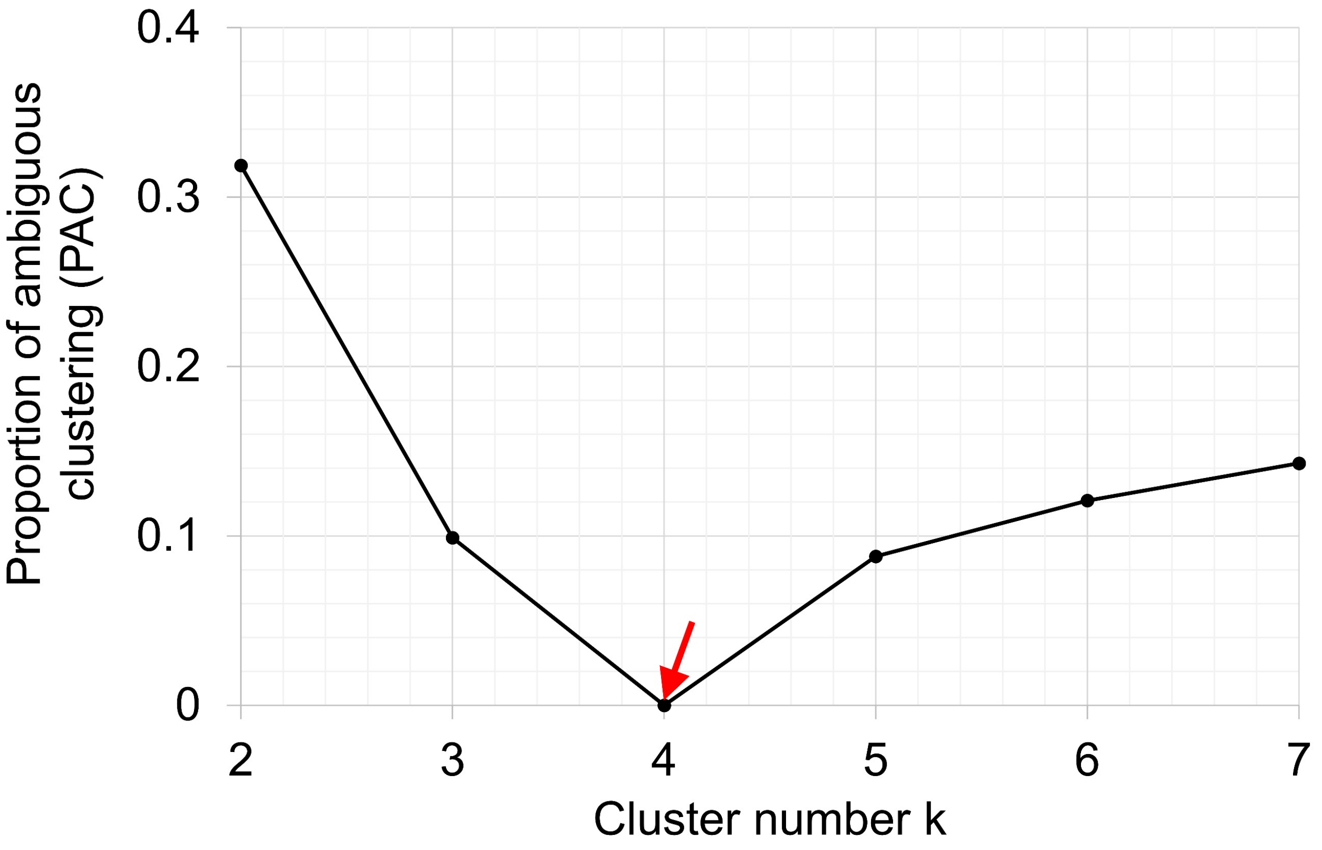

Supplementary Figure S1: *Proportion of ambiguous clustering (PAC) score from correlation matrices from k=1 till k=8 in Pearson correlation-based consensus clustering with pairwise complete observation, Ward.D linkage was applied in 1,000 iterations, optimal k was estimated at k=4 (red arrow). For clustering log2 transformed, column median normalized, and centered matrix was used. Outliers were excluded prior to clustering.*

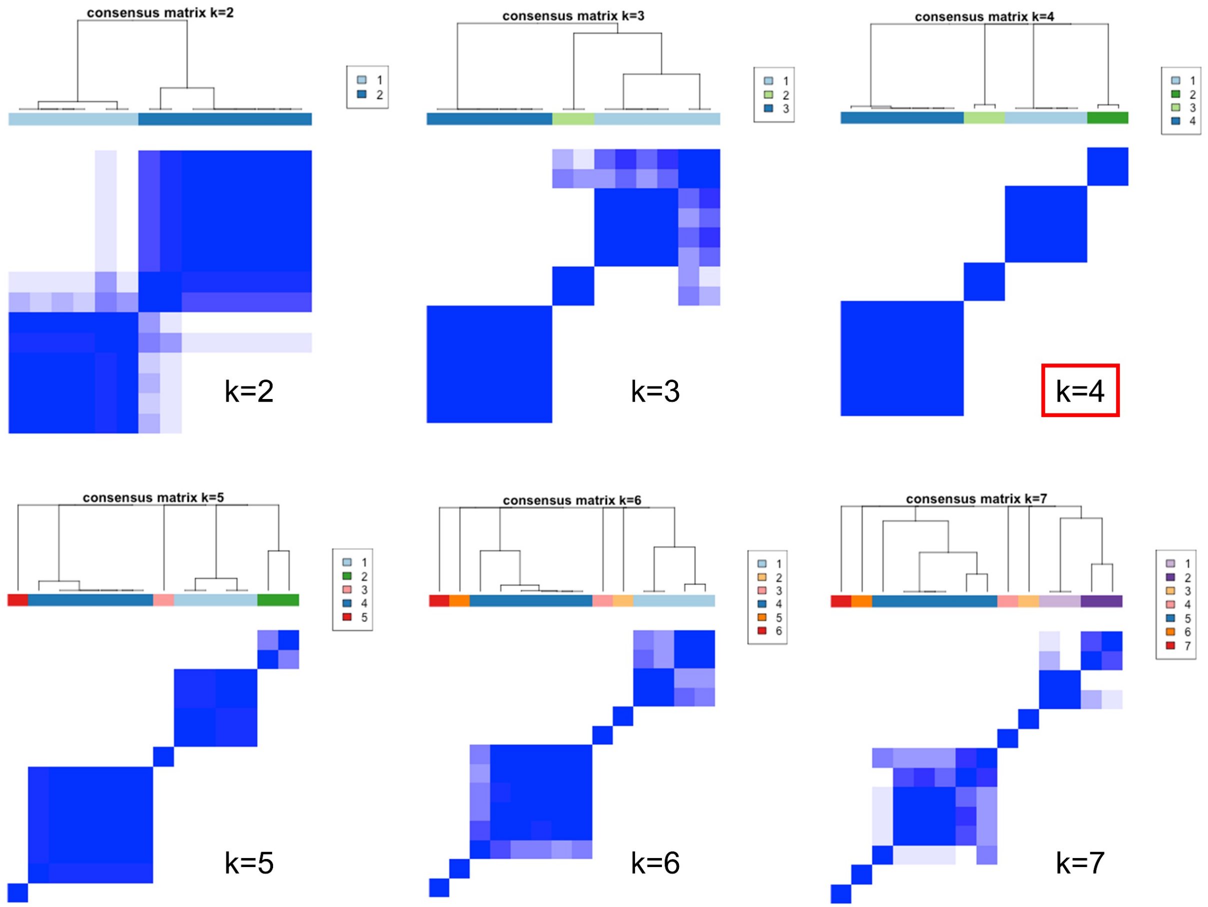

Supplementary Figure S2: *Heatmap visualization of consensus matrices from k=1 to k=7. Consensus values range10 from 0 (never clustered together; white) to 1 (always clustered together; dark blue). Pearson correlation-based11 consensus clustering with pairwise complete observation, Ward.D linkage was applied in 1,000 iterations, Optimal k was estimated at k=4 (red box). For clustering log2 transformed, column median normalized, and centered matrix was used. Outliers were excluded prior to clustering.*

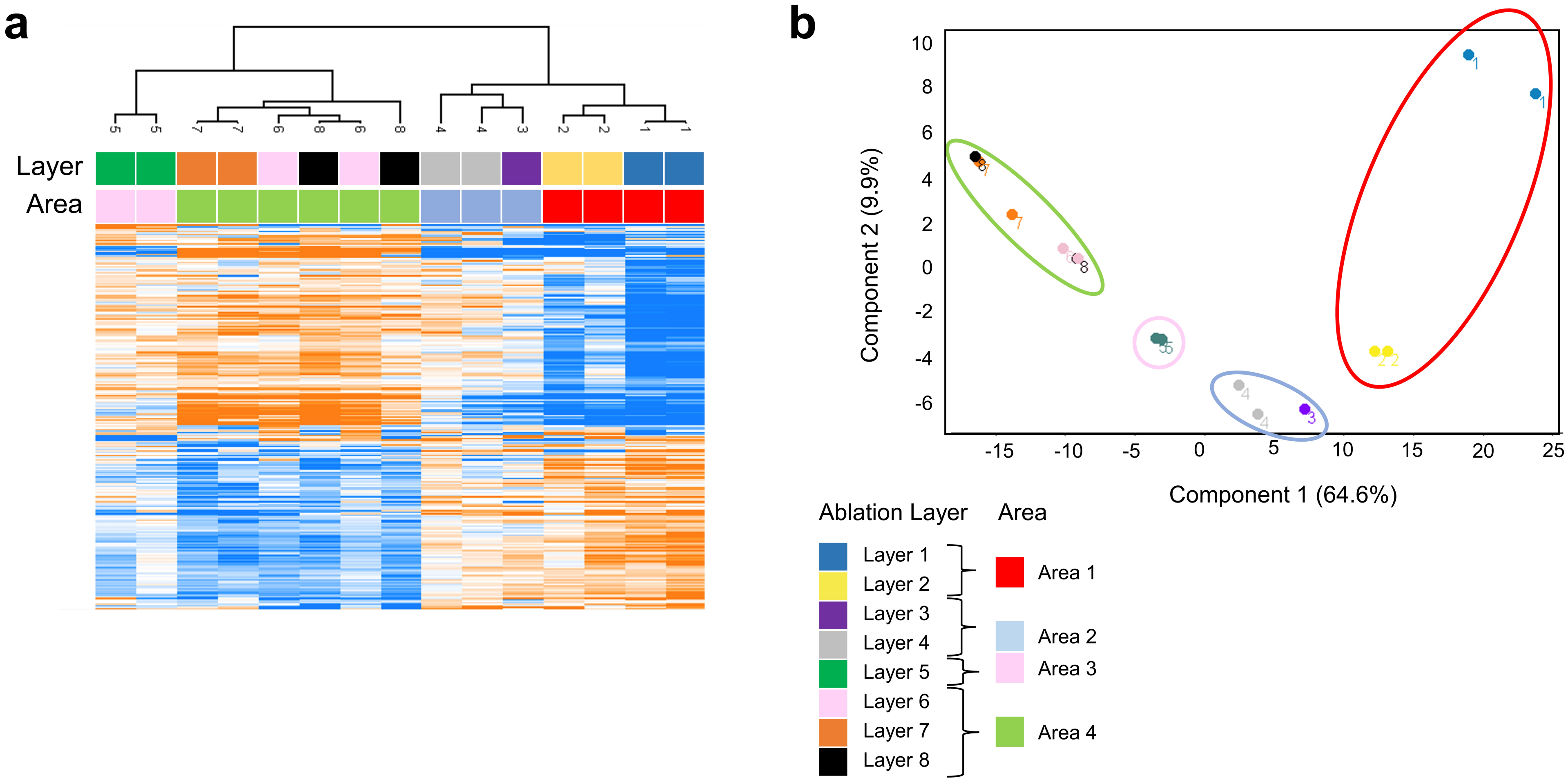

Supplementary Figure S3: *(a) Hierarchical clustering based on 251 ANOVA significant proteins between respective layers. (b) Scatter plot visualization of the first two principal components from PCA, based on 251 ANOVA significant proteins between respective layers.*

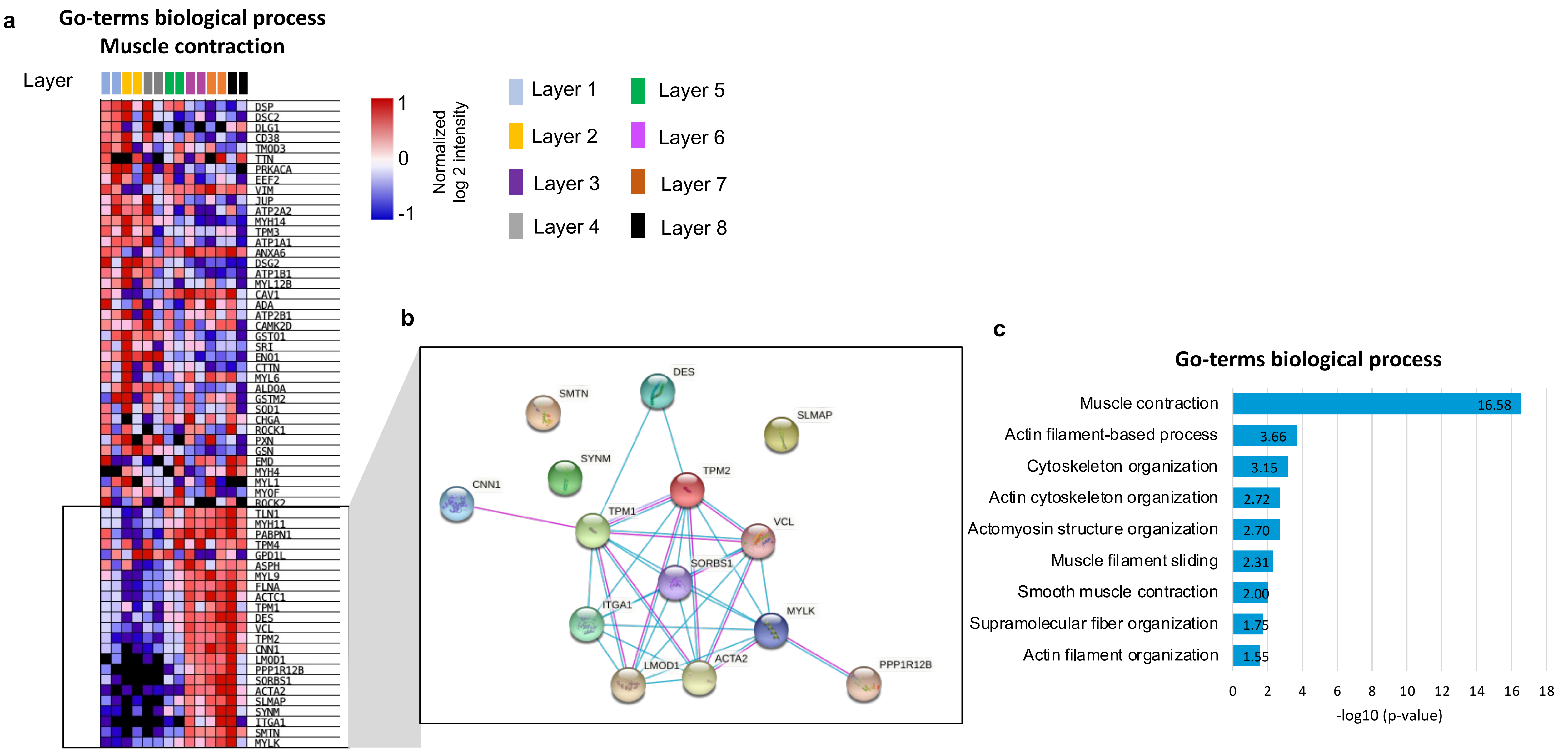

***Supplementary Figure S4:a)** Heatmap visualization of the relative abundance distribution of proteins assigned to the gene set "**Go-terms biological process Muscle contraction**"b) STRING Protein-Protein interaction map of proteins, individually enriched in the Geneset "**Go-terms biological process Muscle contraction**" in gene set enrichment (GSEA). c) Gene-ontology molecular function based enrichment of proteins, individually enriched in the Geneset "**Go-terms biological process Muscle contraction**" in gene set enrichment (GSEA).*
